# 1417. Fosfomycin Use in the Treatment of Complicated Urinary Tract Infections at a Veterans Affairs Medical Center

**DOI:** 10.1093/ofid/ofab466.1609

**Published:** 2021-12-04

**Authors:** Ryan Lee, Thuong Tran, Susanna Tan

**Affiliations:** 1 VA Long Beach, Rancho Palos Verdes, California; 2 Veterans Affairs Long Beach Medical Center, Long Beach, California; 3 VA Long Beach Healthcare System, Long Beach, California

## Abstract

**Background:**

The prevalence of multidrug resistant gram-negative urinary tract infections (UTIs) is increasing, often requiring intravenous antimicrobial therapy. Oral fosfomycin is a recommended alternative agent for the treatment of cystitis caused by extended spectrum beta-lactamase (ESBL)-producing *Escherichia coli* (E. coli). The primary objective of this study is to evaluate the efficacy of fosfomycin in the treatment of UTIs at the Veterans Affairs Long Beach Healthcare System. The secondary objective is to assess the incidence of adverse drug reactions associated with fosfomycin.

**Methods:**

This is a retrospective, single-center, cohort study. Patients who received fosfomycin between June 1^st^, 2015 – June 30^th^, 2020 were included. Data collection was completed by chart review through the Computerized Patient Record System (CPRS). Descriptive analysis was used to evaluate data. Treatment outcomes were analyzed using a composite of clinical and microbiological cure. Clinical cure was defined as resolution of UTI symptoms. Microbiological cure was defined as urine sterilization within 1 month after completing treatment course with fosfomycin.

**Results:**

A total of 62 unique patients were evaluated in this study. The mean age was 71.9 years. 56 patients (90.3%) were male, 31 patients (50.0%) had an indwelling catheter present at the time of treatment, and 48 patients (77.4%) had the presence of genitourinary tract pathology that may increase the risk of developing UTIs. Majority of patients (50%) had a urine culture result positive for *E. coli* prior to treatment, of which 43.5% were ESBL-producing. 60 patients (96.8%) received more than 1 dose of Fosfomycin. Out of 29 patients who were eligible to be evaluated for clinical outcomes, 20 patients (68.9%) met a positive composite outcome of either microbiological cure, clinical cure, or both. 4 patients (6.5%) experienced an adverse drug reaction of diarrhea that was self-limited.

**Conclusion:**

Fosfomycin is an effective and well-tolerated antimicrobial agent that may be considered for treatment of complicated UTIs without evidence of pyelonephritis or bacteremia caused by multi-drug resistant organisms in the veteran population.

**Disclosures:**

**All Authors**: No reported disclosures

